# The consequence of simultaneous and sequential coinfections of *Aedes aegypti* with DENV-1, DENV-2, and CHIKV on vector competence

**DOI:** 10.3389/fcimb.2026.1891568

**Published:** 2026-10-01

**Authors:** Rêgila Mello do Nascimento, Ana Paula Alvim Pereira França Baptista, Alexia Katlen Ribeiro Ramos da Silva, Raquel Soares Maia Godoy, Lucas Henrique-Gomes, Thaís Bonifácio Campolina, Rafael Nacif-Pimenta, Paulo Filemon Paolucci Pimenta, Nágila Francinete Costa Secundino

**Affiliations:** 1 Laboratory of Medical Entomology, Oswaldo Cruz Foundation, Rene Rachou Institute (Fiocruz/Minas), Belo Horizonte, Brazil; 2 Programa de Pós-graduação em Ciências da Saúde, Rene Rachou Institute (Fiocruz/Minas), Belo Horizonte, Brazil; 3 Wesleyan Imaging Facility, Wesleyan University, Middletown, CT, United States

**Keywords:** *Aedes aegypti*, arbovirus coinfection, chikungunya virus, dengue virus serotypes, sequential infection, simultaneous infection, vector competence

## Abstract

Vector-borne diseases (VBD) caused by arboviruses represent a major public health threat in tropical regions. According to WHO’s Global Vector Control Response, more than 80% of the global population live in areas at risk for VBD. For instance, *Aedes aegypti* is the primary vector of dengue virus (DENV) and chikungunya virus (CHIKV), and its widespread distribution, particularly in Brazil, contributes to the occurrence of outbreaks. During these outbreaks, mosquitoes may acquire either monoinfections or coinfections, which can occur either simultaneously or sequentially, potentially influencing transmission dynamics. In this study, we investigated the effects of simultaneous coinfection with CHIKV and two DENV serotypes (DENV-1 and DENV-2) on the vector competence of *Ae. aegypti* and described the characteristics of infection and transmission phenotypes following different orders of sequential arbovirus acquisition. Simultaneous coinfection resulted in serotype-dependent outcomes. At 7 days post-infection (dpi), DENV-1 infection rates and viral loads were significantly reduced compared with monoinfections, whereas DENV-2 showed increased viral loads in saliva. At 14 dpi, CHIKV infection rates were significantly reduced in both the CHIKV+DENV-1 (CD1) and CHIKV+DENV-2 (CD2) groups, whereas CHIKV viral loads were reduced only in the CD2 group. In the sequential coinfection experiments, DENV-2 exhibited lower replication levels than DENV-1 in both sequential infection scenarios, regardless of whether DENV was acquired before or after CHIKV. Likewise, DENV-2 showed lower transmission efficiency than DENV-1, and murine transmission assays confirmed reduced viral loads for DENV-2. Overall, our findings demonstrate that CHIKV–DENV coinfection in *Ae. aegypti* is associated with distinct infection and transmission outcomes depending on the DENV serotype and the infection scenario. In sequentially exposed mosquitoes, distinct phenotypes were observed according to the order of arbovirus acquisition. These results highlight the complexity of arboviral coinfections within this shared vector and emphasize the importance of considering different coinfection scenarios when evaluating vector competence and transmission risk in endemic settings.

## 1 Introduction

Vector-borne diseases (VBDs) are caused by parasites, viruses, and bacteria and are transmitted to humans and other vertebrates through the bite of infected blood-sucking arthropods, such as mosquitoes, ticks, and sandflies. Of the 1, 407 recognized species of human pathogens, 177 are classified as emerging or re-emerging, with approximately 73% of these being arboviruses transmitted by mosquitoes (Mbaoma et al., 2025). It is estimated that nearly 80% of the world’s population lives in areas at risk of exposure to at least one VBD, which collectively are responsible for more than 700, 000 deaths annually (Madewell, 2020; Manouana et al., 2024; World Health Organization, 2024a).

Dengue virus (DENV) and Chikungunya virus (CHIKV) are primarily transmitted by mosquitoes of the genus *Aedes*, particularly *Aedes aegypti* the principal urban vector in tropical and subtropical regions worldwide. However, *Aedes albopictus* has emerged as an important secondary vector, contributing to the maintenance and geographic expansion of both arboviruses, particularly in temperate regions and areas where *Ae. aegypti* is absent or less abundant (Bezerra et al., 2016; Resck et al., 2024). Among the 134 arboviruses known to cause disease in humans, DENV and CHIKV are among the most prevalent and clinically important (Gubler, 2001). DENV and CHIKV belong to the genera *Orthoflavivirus* (formerly *Flavivirus*) and *Alphavirus*, respectively (Postler et al., 2023).

Historical accounts indicate that China first documented dengue as a disease as early as 265–420 CE. More reliable records of epidemics in North America, Asia, and Africa emerged between 1779 and 1780. The growth of international trade and transportation in the 19^th^ century facilitated the global spread of dengue, particularly through port towns, and its incidence continued to increase during the 20^th^ century. In contrast, chikungunya was first recognized during an outbreak in Tanzania in 1952, when CHIKV was subsequently isolated and identified. Today, both diseases are widespread worldwide (Lumsden, 1955; World Health Organization, 2024b).

While outbreaks of diseases with clinical manifestations compatible with dengue have been reported for centuries, the first isolations of DENV-1 and DENV-2 serotypes occurred in 1943 in Japan and 1945 in Hawaii, respectively (Hotta, 1952)., DENV is currently classified into four serotypes (DENV-1 to DENV-4), which share approximately 65% ​​genomic sequence identity and are endemic in more than 100 countries (Messina et al., 2014; Sirisena et al., 2021). CHIKV was first isolated for the first time in 1952 in Tanzania, Africa, and has since spread worldwide (Lumsden, 1955; World Health Organization, 2024b). In Brazil, the circulation of DENV has been documented since the 1980s, with outbreaks recurring every two to five years (Brathwaite Dick et al., 2012; Salles et al., 2018). CHIKV was introduced into Brazil in 2014, resulting in regional outbreaks, particularly in the southeast of the country (Almeida et al., 2023).

*Aedes aegypti*, the primary vector of DENV and CHIKV, is currently widespread throughout Brazil; a distribution driven by poor urban infrastructure, population growth, and socioeconomic inequality (Torres et al., 2024). Additionally, factors such as increased urban mobility, climate and environmental changes, and the lack of effective vector control policies have contributed to the expansion of this vector and, consequently, to the increasing incidence of arboviral diseases (Ministério da Saúde, 2025). According to official surveillance data from Brazil, there were approximately 6 million dengue cases in 2024, compared with approximately 1.5 million cases in 2025. Similarly, approximately 230, 000 chikungunya cases were reported in 2024, decreasing to approximately 100, 000 cases in 2025 (Ministério da Saúde, 2024, 2025).

The coexistence of multiple arboviruses in endemic areas raises concerns about coinfections in both humans and vectors. Although CHIKV-affected areas often overlap with regions endemic for DENV (Mackenzie et al., 2001), the rarity of simultaneous outbreaks raises the question of whether such events are truly uncommon or simply underreported. Nevertheless, confirmed cases of DENV and CHIKV coinfection in humans have been reported in Brazil (Torres et al., 2024), Madagascar (Ratsitorahina et al., 2008), Gabon, Central Africa (Caron et al., 2012), the Caribbean (Omarjee et al., 2014) and the Colombia**–**Venezuela border region (Carrillo-Hernández et al., 2018).

Coinfection can occur by two mechanisms: (i) simultaneous infection with multiple viruses through a single mosquito bite, or (ii) sequential infection, in which the host receives infective bites from mosquitoes infected with different arboviruses (Miralles et al., 2001; Chaves et al., 2018). Studies have reported that *Ae. aegypti* can simultaneously transmit Zika virus (ZIKV) and CHIKV through its saliva, with transmission rates as high as 12% for both viruses (Göertz et al., 2017). In Brazil, *Ae. aegypti* populations from Manaus are highly permissive to DENV/ZIKV coinfections and can transmit both pathogens during a single bite, with increased transmission efficiency observed for ZIKV to vertebrate hosts (Chaves et al., 2018). Furthermore, the dynamics of dual or triple coinfections can alter vector competence and the viral abundance of one or more viruses (Rodrigues et al., 2021). Evidence for the occurrence of coinfections in nature is provided by reports of vertical transmission from female mosquitoes to their offspring, resulting in larvae simultaneously infected with DENV and ZIKV (Teixeira et al., 2021).

Sequential coinfections, in which a mosquito already infected with one arbovirus is later exposed to another, are considered more likely to occur than simultaneous coinfections. Importantly, the interactions between the vector and each virus under these conditions can differ substantially, sometimes leading to unexpected effects on vector competence (Peng et al., 2024). Primary arbovirus infections can modulate vector susceptibility to subsequent infections by influencing viral replication, dissemination, and transmission efficiency. Consequently, these interactions may alter viral abundance and vector competence, thereby influencing the circulation of co-circulating arboviruses and the dynamics of outbreaks in endemic regions (Rodrigues et al., 2021). In the case of DENV and ZIKV, an initial DENV infection has been shown to reduce ZIKV dissemination in C6/36 cells and in *Ae. aegypti* (Rückert et al., 2017; Peng et al., 2024). Conversely, synergistic interactions have also been reported. *Ae. aegypti* infected with CHIKV either before or after exposure to ZIKV exhibited increased ZIKV transmission at seven days after the second infection (Magalhaes et al., 2018). Similarly, prior ZIKV infection increased the infection rate of Mayaro virus (MAYV) in *Ae. aegypti* (Brustolin et al., 2023).

In natural settings, mosquitoes are frequently coinfected with multiple arboviruses, and human coinfections have also been increasingly reported. The complex dynamics of coinfection and cotransmission pose significant challenges to public health, particularly in regions where multiple arboviruses co-circulate, potentially increasing the incidence and severity of outbreaks. Understanding the dynamics of coinfections and their implications for the vector competence of *Ae. aegypti* is crucial for understanding arbovirus transmission and for guiding vector surveillance and control strategies, particularly in endemic regions where multiple arboviruses coexist. In Brazil, surveillance methods such as the Rapid Index Survey for *Ae. aegypti* (LIRAa/LIA) are used to estimate vector infestation and outbreak risk; however, these tools do not assess mosquito coinfections (Vogels et al., 2019; Teixeira et al., 2021).

In this study, we experimentally coinfected *Ae. aegypti* with CHIKV and two dengue virus serotypes (DENV-1 and DENV-2) under simultaneous and sequential infection protocols. We investigated the consequences of simultaneous coinfection on vector competence and characterized infection and transmission phenotypes following different orders of sequential arbovirus acquisition. Given the known serotype-specific differences in DENV replication, we focused on DENV-1 and DENV-2. This study was motivated by the intense cocirculation of dengue and chikungunya observed in Belo Horizonte during the 2024 outbreak, when 216, 680 dengue and 10, 815 chikungunya cases were reported (Ministério da Saúde, 2024). The laboratory-maintained *Ae. aegypti* colony and previously characterized viral isolates were used as experimental models to investigate these epidemiologically relevant coinfection scenarios. A better understanding of mosquito–arbovirus interactions and the infection and transmission phenotypes associated with different coinfection scenarios is important for understanding arbovirus transmission dynamics and advancing vector surveillance and control (Salas-Benito and De Nova-Ocampo, 2015; Vogels et al., 2019).

## 2 Materials and methods

### 2.1 Ae. aegypti colony

A well-established Brazilian colony of *Ae. aegypti* (strain BH), maintained at the Laboratory of Medical Entomology, Fiocruz-Minas, Brazil, was used in this study. Mosquito eggs were collected using ovitraps in the city of Belo Horizonte, Minas Gerais, Brazil, in 2019. Mosquitoes were reared and maintained under standard insectary conditions at 27 °C, with 80% relative humidity, and a (16-h light/8-h dark photoperiod).

### 2.2 DENV-1, DENV-2 and CHIKV

The arboviruses CHIKV (GenBank: MH823668) (Vasconcellos et al., 2019), DENV-1, (GenBank: KP188540) and DENV-2 (GenBank: KP188569; https://www.ncbi.nlm.nih.gov/nuccore/KP188569) were maintained and propagated in C6/36 cells cultured in Leibovitz’s L-15 medium supplemented with 2% heat-inactivated fetal bovine serum (FBS), 5 μg/mL amphotericin B, 100 U/mL penicillin, and 100 μg/mL streptomycin (White, 1987). Virus titration was performed in Vero cells using a plaque assay (LaBarre and Lowy, 2001).

### 2.3 Experimental infections in *Ae. aegypti*

#### 2.3.1 Monoinfection and simultaneous coinfection

Three- to five-day-old female *Aedes aegypti* mosquitoes were divided into five experimental groups (n = 200 per group) and infected by membrane feeding (Secundino et al., 2017). The infectious blood meal was prepared by mixing mouse blood and virus suspension at a 2:1 (v/v) ratio. The final blood meal contained 1 × 10^5^ PFU/mL of virus. For the simultaneous coinfection groups, CHIKV and DENV were each present at the same final infectious titer (1 × 10^5^ PFU/mL per virus).

The following experimental groups of infected *Ae. aegypti* were established: (i) DENV-1 (D1); (ii) DENV-2 (D2); (iii) CHIKV (C); (iv) CHIKV+DENV-1 (CD1); and (v) CHIKV+DENV-2 (CD2). After feeding, engorged females were maintained on 10% glucose *ad libitum* for 21 days post-infection (dpi).

#### 2.3.2 Sequential coinfection

Three- to five-day-old female *Ae. aegypti* mosquitoes were divided into four experimental groups (n = 300 per group) and infected by membrane feeding as described above. The sequential coinfection assay was designed to simulate potential sequential arbovirus exposure scenarios in nature and characterize infection and transmission outcomes according to the order of virus acquisition. For the first infection, one group received DENV-1, another received DENV-2, and two groups received CHIKV. Seven days later, mosquitoes were exposed to a second blood meal containing the corresponding second virus as follows: (i) DENV-1 followed by CHIKV (D1C); (ii) DENV-2 followed by CHIKV (D2C); (iii) CHIKV followed by DENV-1 (CD1); and (iv) CHIKV followed by DENV-2 (CD2). After the second blood meal, engorged females were maintained on 10% glucose *ad libitum* for 14 days post-coinfection (dpci).

#### 2.3.3 Blood collection for mosquito infection assays

For the mosquito infection assays described in this section, fresh blood was obtained from BALB/c mice by cardiac puncture. Animals were anesthetized with an intraperitoneal injection of ketamine (200 mg/kg) and xylazine (10 mg/kg). Blood was collected immediately after surgical anesthesia had been confirmed. Following blood collection, mice were euthanized by intraperitoneal administration of sodium thiopental (150 mg/kg), administered as an overdose (three times the anesthetic dose).

### 2.4 RNA extraction and virus quantification

For the monoinfection groups (DENV-1, DENV-2, and CHIKV) and the simultaneous coinfection groups (CD1 and CD2): 30 mosquitoes per group were randomly selected at 7, 14, and 21 dpi. For the sequential coinfection groups (CD1, CD2, D1C, and D2C), 30 mosquitoes were also randomly selected at 7 and 14 dpci. The selected mosquitoes were individually transferred into separate microtubes. Viral RNA was extracted using the QIAamp Viral RNA Mini Kit (Qiagen, Hilden, Germany) according to the manufacturer’s instructions and stored at −70 °C. Viral RNA quantification was performed using quantitative RT-qPCR (7500 Fast Real-Time PCR, Applied Biosystems) using the TaqMan Fast Virus 1-Step kit (Thermo Fisher Scientific). Virus detection was based on specific TaqMan probes and primers, as previously described by Edwards et al. (2007) and Leparc-Goffart et al. (2009).

### 2.5 Saliva collection and transmission efficiency (TE)

TE was assessed at 14 dpi for all monoinfection and simultaneous coinfection groups and at 14 dpci for the sequential coinfection groups. At each time point, 10 mosquitoes from each experimental group were randomly selected for saliva collection. In one independent experiment, fewer than 10 mosquitoes were available for saliva analysis due to mortality following infection. Mosquitoes were cold anesthetized, and their legs and wings were removed. The proboscis of each mosquito was inserted into a filter tip containing 6 μL of a 1:1 mixture of sucrose solution and fetal bovine serum for 30 min. Saliva samples were collected into 1.5 mL tubes containing 100 μL of L-15 medium and stored at −70 °C until processing (do Nascimento et al., 2022). Saliva samples were analyzed using RT-qPCR to determine viral RNA loads. Viral load (VL), expressed as the number of viral RNA copies detected in each saliva sample, was used as a measure of infection intensity. Transmission efficiency (TE) was calculated as the proportion of mosquitoes with detectable viral RNA in their saliva relative to the total number of mosquitoes tested.

### 2.6 Transmission by bite of coinfected *Ae. aegypti*

Concurrently with the forced salivation assay, a mosquito bite transmission assay was performed to confirm the presence of infectious virus in saliva and evaluate successful transmission to a vertebrate host during blood feeding. This assay was designed to assess virus transmission to the vertebrate host rather than infection status in individual mosquitoes. To increase the chances of successful blood feeding and virus transmission, ten female mosquitoes were used per mouse. Transmission by bite was evaluated at 14 dpci in the sequential coinfection groups (CD1, CD2, D1C, and D2C). For each experimental group, ten female *Ae. aegypti* mosquitoes were divided into two pools of five females. Each pool was transferred to a 3-dram plastic vial (4.8 cm in height × 1.8 cm in diameter) covered with 0.25-mm nylon mesh. One vial was placed against each ear of an anesthetized BALB/c mouse, and mosquitoes were allowed to feed for 1 h (Secundino et al., 2017).

BALB/c mice were anesthetized with ketamine (200 mg/kg) and xylazine (10 mg/kg). After adequate anesthesia was confirmed, animals were positioned in a custom restraint device with both ears exposed for mosquito feeding. At the end of the feeding period, mice were euthanized by intraperitoneal administration of sodium thiopental (150 mg/kg). Death was confirmed by the absence of respiratory movements and cardiac activity before tissue collection. A 4-mm punch biopsy was then collected from the mosquito-exposed area of each ear, and the right and left ear samples were transferred individually to microtubes for RNA extraction and RT-qPCR, as previously described.

### 2.7 Statistical analysis

Three independent biological experiments were performed under identical experimental conditions for both the monoinfection and the simultaneous and sequential coinfection assays. Inter-experiment variability was assessed using the Kruskal–Wallis test. As no significant differences were detected among the independent biological experiments (p > 0.05), data were pooled for the final statistical analyses. Data distribution normality was assessed using the Shapiro–Wilk test. Because viral load data did not follow a Gaussian distribution, comparisons between groups were performed using the Wilcoxon–Mann–Whitney test. Differences in infection rates (IR) and transmission efficiency (TE) were evaluated using the two-tailed χ² test or Fisher’s exact test. Correlations among arbovirus cDNA copy numbers were assessed using Spearman’s rank correlation test. All statistical analyses were performed using GraphPad Prism version 7.00 (GraphPad Software, La Jolla, CA, USA), and statistical significance was set at p < 0.05.

## 3 Results

### 3.1 Impact of simultaneous coinfection on the vector competence of *Ae. aegypti* for DENV and CHIKV

To assess the effects of simultaneous coinfection on vector competence, we compared monoinfected and simultaneously coinfected mosquitoes using infection rates (IR), viral loads in mosquito bodies at 7, 14, and 21 dpi, and transmission efficiency (TE) and saliva viral loads at 14 dpi. To further characterize the infection status of mosquitoes within the simultaneous coinfection groups, individual mosquitoes were classified according to the detection of DENV and CHIKV as positive for both viruses, positive only for DENV, positive only for CHIKV, or negative for both. Dual infection was the predominant outcome in both coinfection groups at most time points evaluated. At 21 dpi, dual positivity remained high in the DENV-1/CHIKV group (96.7%), whereas in the DENV-2/CHIKV group, 53.3% of mosquitoes were positive for both viruses and 46.7% were positive only for CHIKV. A small percentage of mosquitoes were positive for only one virus, whereas only one mosquito (from the DENV-2/CHIKV group at 14 dpi) was negative for both viruses (**Supplementary Table 1**).

In the DENV-1 monoinfected group (D1), the IR was 100%, 97%, and 100% at 7, 14, and 21 dpi, respectively, whereas in the CHIKV+DENV-1 simultaneous coinfection group (CD1), IR was 87%, 90%, and 100% at the corresponding time points. At 7 dpi, the DENV-1 IR was significantly lower in the CD1 group than in the monoinfection group (p = 0.038), whereas no significant differences were observed at 14 or 21 dpi (**Figures 1A–C**). Median viral loads in the D1 group were 7.14 × 10^6^, 1.25 × 10^7^, and 3.75 × 10^7^ viral cDNA copies at 7, 14, and 21 dpi, respectively, compared with 1.17 × 10^4^, 2.58 × 10^4^, and 6.75 × 10³ copies in the CD1 group. A significantly lower viral load was observed in the CD1 group at 7 dpi (p = 0.0017), whereas no significant differences were detected at 14 or 21 dpi (**Figures 1A–C**). At 14 dpi, TE was 70% in the D1 group and 65% in the CD1 group, with median saliva viral loads of 3.29 × 10² and 7.44 × 10³ copies, respectively. No significant difference in TE was observed between the two groups (**Figure 1D**).

**Figure 1:**
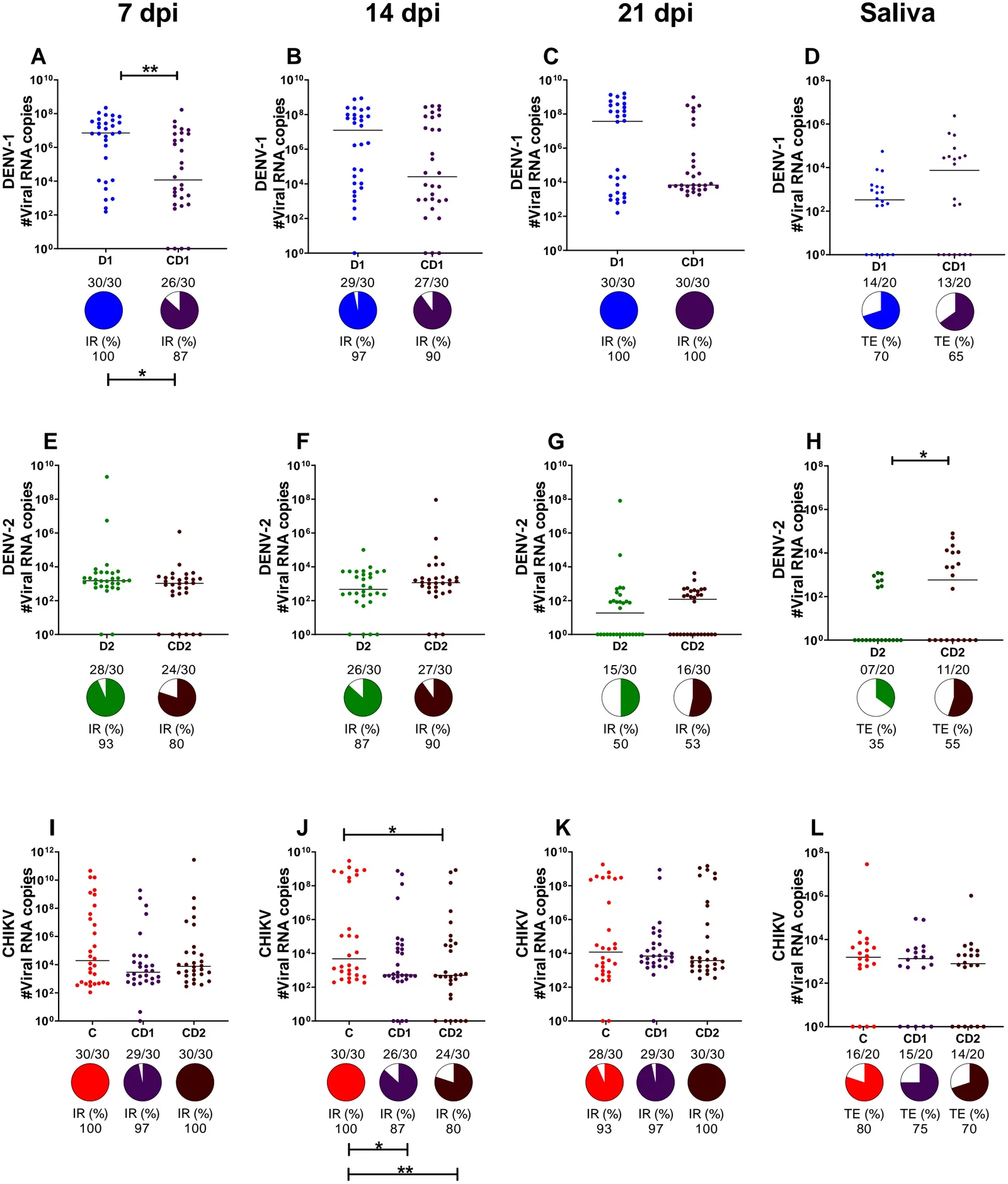
Viral loads of *Ae. aegypti*-BH following monoinfection or simultaneous coinfection with DENV-1, DENV-2, and CHIKV at 7, 14, and 21 days post-infection (dpi). Viral loads are shown by experimental group, tissue, and time point. **(A–D)** DENV-1 viral load. **(E–H)** DENV-2 viral load. **(I–L)** CHIKV viral load. Each color represents a different infection condition: blue, DENV-1 monoinfection; green, DENV-2 monoinfection; red, CHIKV monoinfection; purple, DENV-1 + CHIKV coinfection; and brown, DENV-2 + CHIKV coinfection. Pie charts below each group show the infection rate of mosquitoes for each condition, while those corresponding to saliva indicate transmission efficiency. Data represent three independent biological experiments pooled for statistical analysis. Each dot represents an individual mosquito, and horizontal lines indicate the median. Statistical significance is indicated by one (*) or two (**) asterisks corresponding to p ≤ 0.05 and p ≤ 0.01, respectively.

In the DENV-2 monoinfection group (D2), IR was 93%, 87%, and 50% at 7, 14, and 21 dpi, respectively, whereas in the CHIKV+DENV-2 simultaneous coinfection group (CD2), IR was 80%, 90%, and 53% at the corresponding time points. No significant differences in IR were observed between the groups (**Figures 1E–G**). Median viral loads in the D2 group were 1.51 × 10³, 4.69 × 10², and 1.08 × 10¹ cDNA copies at 7, 14, and 21 dpi, respectively, compared with 1.05 × 10³, 1.16 × 10³, and 1.21 × 10² cDNA copies in the CD2 group. No significant differences in viral loads were detected at any time point (**Figures 1E–G**). At 14 dpi, transmission efficiency (TE) was 35% (7/20 saliva-positive mosquitoes) in the D2 group and 55% in the CD2 group. Although TE did not differ significantly between groups, the median saliva viral load was significantly higher in the CD2 group (5.81 × 10² cDNA copies) than in the monoinfection group (p = 0.0390) (**Figure 1H**).

In the CHIKV monoinfected group (C), IR was 100% at 7 and 14 dpi, and 93% at 21 dpi. Median viral loads were 1.93 × 10^4^, 4.77 × 10³, and 1.19 × 10^4^ cDNA copies at the corresponding time points (**Figures 1I–K**). At 14 dpi, TE was 80%, with a median saliva viral load of 1.54 × 10³ cDNA copies (**Figure 1L**).

In the DENV-1 + CHIKV simultaneous coinfection group (CD1), CHIKV IR was 97% at 7 dpi, 87% at 14 dpi, and 97% at 21 dpi, with median viral loads of 2.90 × 10³, 5.43 × 10², and 6.86 × 10³ cDNA copies, respectively. Compared with the monoinfected group, CHIKV IR was significantly lower at 14 dpi (p = 0.038), whereas no significant differences in viral load were detected (**Figures 1I–K**). TE was 75%, with a median saliva viral load of 1.34 × 10³ cDNA copies, which did not differ significantly from the monoinfected group (**Figure 1L**).

In the DENV-2 + CHIKV simultaneous coinfection group (CD2), CHIKV IR reached 100% at 7 dpi, 80% at 14 dpi, and 100% at 21 dpi, with median viral loads of 7.61 × 10³, 4.98 × 10², and 3.74 × 10³ cDNA copies, respectively (**Figures 1I–K**). At 14 dpi, both CHIKV IR (p = 0.0098) and viral load (p = 0.0166) were significantly lower than those in the CHIKV monoinfected group (**Figure 1J**). TE was 70%, with a median saliva viral load of 7.77 × 10² cDNA copies, which did not differ significantly from the CHIKV monoinfected group (**Figure 1L**).

### 3.2 DENV-1 and DENV-2 infection and transmission phenotypes following sequential exposure with CHIKV

To characterize DENV infection and transmission phenotypes following sequential arbovirus exposure, we measured DENV-1 and DENV-2 infection rates, viral loads, and transmission efficiency in mosquitoes exposed to each DENV serotype before or after CHIKV. These analyses allowed us to stratify DENV outcomes by serotype and order of virus acquisition.

In the CD1 group, DENV-1 IR was 100% at both 7 and 14 dpci. In the CD2 group, DENV-2 IR was 93% at both time points. Median viral loads differed between the two groups at both time points (**Figures 2A, B**). At 7 dpci, viral loads were 7.54 × 10² cDNA copies in CD1 and 2.27 × 10^4^ in CD2 (p < 0.0001). At 14 dpci, median viral loads were 4.16 × 10³ versus 1.57 × 10³ cDNA copies, respectively (p = 0.007) (**Figures 2A, B**). At 14 dpci, TE was 55% for DENV-1 and 65% for DENV-2, with viral loads of 1.40 × 10² and 2.28 × 10² cDNA copies, respectively (**Figure 2C**). Additionally, the ability of *Ae. aegypti* females to transmit the virus to vertebrate hosts by bite was assessed using BALB/c mice. In this assay, TE was 20% for DENV-1 and 10% for DENV-2 (**Figure 2D**).

**Figure 2:**
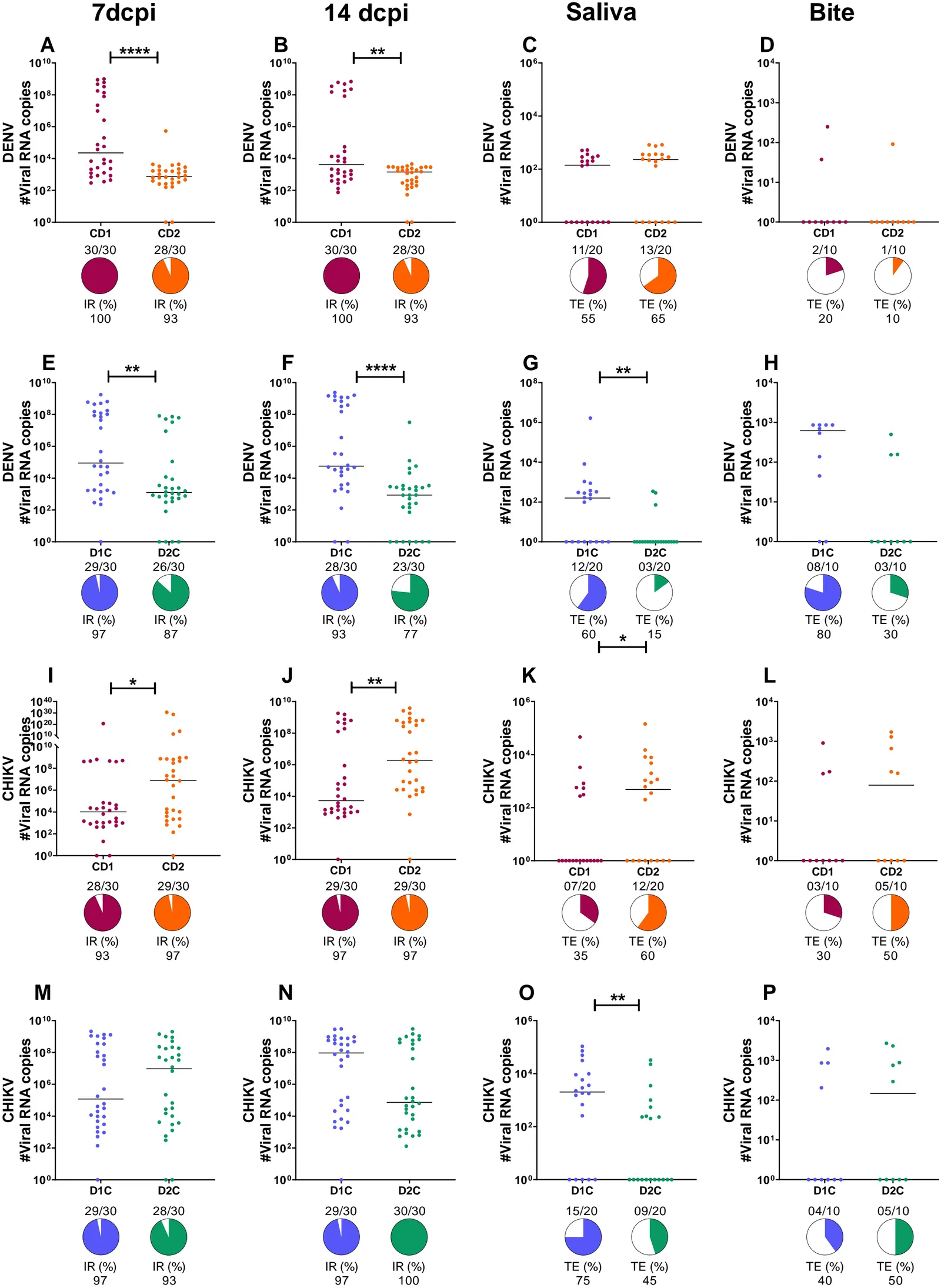
DENV viral loads in mosquitoes sequentially coinfected with DENV-1 or DENV-2 and CHIKV. Viral loads were quantified in mosquitoes, saliva, and mouse ears at 7 and 14 days post-coinfection (dpci). **(A–D)** Comparison of DENV viral loads in groups that received CHIKV as the first infection (CD1 and CD2). **(E–H)** Comparison of DENV viral loads between groups initially infected with DENV-1 or DENV-2 (D1C and D2C). **(I–L)** Comparison of CHIKV viral loads in groups that received CHIKV as the first infection (CD1 and CD2). **(M–P)** Comparison of CHIKV viral loads between groups initially infected with DENV-1 or DENV-2 (D1C and D2C). The order of letters indicates the sequence of infections. Pie charts below each group show the infection rate for the corresponding experimental group, whereas pie charts corresponding to saliva show transmission efficiency. Data represent three independent biological experiments pooled for statistical analysis. Each dot represents an individual mosquito, and horizontal lines indicate the median. Statistical significance is indicated by one (*), two (**), three (***) or four (****) asterisks, corresponding to p ≤ 0.05, p ≤ 0.01, p ≤ 0.001, and p ≤ 0.0001, respectively.

In the sequential coinfections established with prior DENV infection, mosquitoes were first exposed to DENV-1 or DENV-2 and subsequently to CHIKV (D1C and D2C). The D1C group exhibited DENV-1 infection rates of 97% and 93% at 7 and 14 dpci, respectively, whereas the D2C group showed DENV-2 infection rates of 87% and 77% at the corresponding time points (**Figures 2E, F**). As observed in the CHIKV/DENV sequence, DENV-2 exhibited significantly lower viral loads than DENV-1. At 7 dpci, median viral loads were 1.87 × 10³ cDNA copies for D2C and 1.19 × 10^5^ for D1C (p = 0.0013). At 14 dpci, median viral loads were 2.20 × 10³ and 1.92 × 10^5^ cDNA copies, respectively (p < 0.0001) (**Figures 2E, F**). At 14 dpci, TE was 60% for DENV-1 and 15% for DENV-2. Saliva viral loads were significantly lower for DENV-2 than for DENV-1 (p = 0.0024), with a median viral load of 1.59 × 10² cDNA copies detected in D1C, whereas only 3/20 saliva samples were positive in D2C (**Figure 2G**). In the mouse transmission assay, TE was 80% for DENV-1 and 30% for DENV-2. Likewise, DENV-2 exhibited significantly lower viral loads than DENV-1 (p = 0.01), with a median viral load of 6.16 × 10² cDNA copies in D1C, whereas only 3/10 ear tissue samples were positive in D2C (**Figure 2H**).

### 3.3 CHIKV infection and transmission phenotypes after sequential exposure to DENV-1 or DENV-2

Next, we characterized CHIKV infection and transmission phenotypes in the same sequential exposure groups by evaluating infection rates, viral loads, and transmission efficiency according to the order of virus acquisition and the DENV serotype used in each exposure sequence.

In the sequential coinfections initiated with prior CHIKV infection, mosquitoes were first exposed to CHIKV and subsequently challenged with DENV-1 (CD1) or DENV-2 (CD2). The CD1 group exhibited CHIKV infection rates (IR) of 93% and 97% at 7 and 14 dpci, respectively, whereas the CD2 group showed IRs of 97% at both time points (**Figures 2I–K**). CHIKV viral loads differed between the CD1 and CD2 groups, with significantly lower values in CD1 at both time points. At 7 dpci, median viral loads were 1.03 × 10^4^ and 7.95 × 10^6^ cDNA copies in CD1 and CD2, respectively (p = 0.0243). At 14 dpci, median viral loads were 5.26 × 10³ and 1.90 × 10^6^ cDNA copies, respectively (p = 0.007) (**Figures 2I, J**). In the saliva transmission assays performed at 14 dpci, CHIKV transmission efficiency (TE) was 35% in CD1 and 60% in CD2, with saliva positivity detected in 7/20 and 12/20 mosquitoes, respectively (**Figure 2K**). In the mouse transmission assays, TE was 30% in CD1 and 50% in CD2, with positive ear tissue samples detected in 3/10 and 5/10 mice, respectively (**Figure 2L**).

In the reciprocal infection sequence, mosquitoes were first exposed to DENV-1 or DENV-2 and subsequently challenged with CHIKV (D1C and D2C). The D1C group exhibited CHIKV infection rates of 97% at both 7 and 14 dpci, whereas the D2C group showed infection rates of 93% and 100% at the respective time points (**Figures 2M, N**). At 7 dpci, median CHIKV viral loads were 1.18 × 10^5^ cDNA copies in D1C and 9.41 × 10^6^ in D2C. At 14 dpci, median viral loads were 9.21 × 10^7^ in D1C and 7.31 × 10^4^ cDNA copies in D2C (**Figures 2M, N**). At 14 dpci, CHIKV transmission efficiency (TE) was 75% in D1C and 45% in D2C. Saliva positivity was observed in 15/20 mosquitoes in D1C and 9/20 mosquitoes in D2C (**Figure 2O**). In the mouse transmission assay, TE was 40% in D1C and 50% in D2C, with positive ear tissue samples detected in 4/10 and 5/10 mice, respectively (**Figure 2P**).

## 4 Discussion

This study examines the transmission dynamics associated with two ecologically relevant coinfection scenarios, namely, simultaneous and sequential coinfection, using two epidemiologically significant arboviruses: dengue virus (DENV) and chikungunya virus (CHIKV). Under monoinfection conditions *in vitro*, DENV-2 has consistently demonstrated elevated viral replication levels relative to DENV-3 (Quintero-Gil et al., 2014; Niu et al., 2020) and DENV-1 (Quintero-Gil et al., 2018). Complementary *in vivo* studies employing *Ae. aegypti* and *Aedes albopictus* infected with the four DENV serotypes have similarly reported enhanced viral loads for DENV-1 and DENV-2 when compared with DENV-3 and DENV-4 (Ekwudu et al., 2020). These serotype-specific differences in viral replication kinetics may contribute to differences in vector competence, transmission efficiency, and overall epidemiological potential and should therefore be considered when interpreting coinfection experiments involving different DENV serotypes.

These serotype-specific differences in viral replication kinetics may significantly modulate vector competence, transmission efficiency, and overall epidemiological potential, particularly in contexts of coinfection with CHIKV.

Under coinfection conditions, the structural and functional distinctions between the genera *Alphavirus* and *Orthoflavivirus* may contribute to different outcomes in the infection and transmission processes of *Ae. aegypti*. Although both viruses share the same vector and are positive-sense single-stranded RNA viruses, they differ in structural features, replication strategies, and interactions with the mosquito immune system. Alphaviruses replicate in the cytoplasm (Laurent et al., 2022; Girard et al., 2024), whereas orthoflaviviruses preferentially establish replication complexes associated with the rough endoplasmic reticulum (RER) (Welsch et al., 2009; Rückert and Ebel, 2018). These biological disparities may influence coinfection dynamics and could partially elucidate the antagonistic or synergistic interactions identified in our study.

In simultaneous coinfection, CHIKV was associated with reductions in both the prevalence and viral load of DENV-1 at 7 dpi. These results suggest that CHIKV was associated with reduced DENV-1 replication when *Ae. aegypti* mosquitoes were co-infected with both viral species. However, this effect did not extend to viral loads in saliva or transmission efficiency, indicating that horizontal transmission to vertebrate hosts was not significantly affected. A potential area for further investigation is the impact of this interaction on vertical transmission of DENV-1, since reduced viral load and prevalence in the mosquito body may limit successful viral passage to progeny during the early post-infection period.

This finding is consistent with studies that demonstrate a similar effect in interactions between CHIKV and ZIKV (Rückert et al., 2017) and between MAYV and ZIKV (Brustolin et al., 2023), in which ZIKV viral load was reduced. Differences in coinfection outcomes are also reflected *in vitro*. In C6/36 cells, when DENV-3 was introduced at a higher multiplicity of infection (MOI = 1.0) than CHIKV (MOI = 0.1), only DENV replication was detected, indicating inhibition of CHIKV replication (Potiwat et al., 2011). Conversely, in Vero and human liver cells, CHIKV replication increased while DENV replication decreased (Zaidi et al., 2018; Taraphdar et al., 2022). These findings emphasize that coinfection dynamics are context-dependent, with outcomes varying by host, cell type, and virus strain. Notably, in mosquitoes, CHIKV viral load also increased during simultaneous coinfection with orthoflaviviruses (Rodrigues et al., 2021).

In contrast to DENV-1, simultaneous CHIKV coinfection did not increase whole-body DENV-2 viral loads but was associated with significantly higher viral loads in saliva. This finding suggests that CHIKV may facilitate DENV-2 dissemination to, or release from, the salivary glands without necessarily increasing systemic viral loads. However, this interpretation should be considered with caution because viral loads were quantified using whole mosquito homogenates rather than dissected salivary glands. Consequently, the present study cannot determine whether the increased salivary viral load resulted from enhanced dissemination to the salivary glands or from greater viral accumulation within this tissue. Nevertheless, elevated salivary viral loads remain epidemiologically relevant because a higher viral inoculum delivered during mosquito feeding is more likely to establish productive infections in vertebrate hosts by increasing the probability of overcoming early innate immune responses (Althouse et al., 2014; Le Coupanec et al., 2017).

An additional observation was the marked reduction in DENV-2 detection at 21 dpi in both the monoinfection and simultaneous coinfection groups. Because independent groups of mosquitoes were analyzed at each sampling time point, this pattern should not be interpreted as evidence of viral clearance within individual mosquitoes. Rather, it indicates that a smaller proportion of mosquitoes had detectable DENV-2 infection at 21 dpi.

At 14 dpi, DENV-1 coinfection was associated with reduced CHIKV prevalence, while DENV-2 coinfection was associated with reductions in both CHIKV prevalence and viral load. These results suggest that both dengue serotypes may negatively influence CHIKV replication and/or establishment during later infection stages. A decrease in prevalence may translate into reduced vertical transmission potential of CHIKV, given that fewer females remain infected and capable of transmitting virus to progeny. In the case of DENV-2, the reduction in CHIKV viral load further strengthens this hypothesis, as lower systemic loads may reduce the likelihood of viral dissemination to the ovaries and subsequent establishment of transovarial infection (Ciota and Kramer, 2010; Vazeille et al., 2010). However, transmission efficiency and CHIKV salivary load remained unaffected, suggesting that coinfection may not substantially alter horizontal transmission from mosquitoes to vertebrate hosts.

An important consideration is that CHIKV transmission to vertebrate hosts may occur considerably earlier than 14 dpi. Previous studies have demonstrated the rapid progression of CHIKV through mosquito tissue barriers and its relatively short extrinsic incubation period, with infectious virus being detected in saliva during the first days after exposure. Moreover, arbovirus infection and transmission are dynamic processes, and salivary gland infection or viral release into saliva may vary substantially over time (Robison et al., 2020; Viginier et al., 2023; Sartsanga et al., 2025). Therefore, because transmission was assessed at a single, relatively late time point in the present study, transient differences in CHIKV transmission between monoinfected and coinfected mosquitoes may not have been detected. Our findings should thus be interpreted as showing no detectable effect on CHIKV transmission at 14 dpi, rather than demonstrating the absence of an effect throughout the extrinsic incubation period. Since both DENV-1 and DENV-2 were associated with reduced CHIKV infection outcomes, biological features shared across dengue serotypes, such as overlapping cell tropism or conserved patterns of immune stimulation, may represent potential explanations that warrant further investigation. Potential mechanisms underlying these viral interactions include: (i) competition for target cells in the midgut or secondary tissues; (ii) competition for cellular and metabolic resources, including the host translational machinery and energy pools; and (iii) modulation of mosquito immune responses, because arboviruses may differentially activate RNA interference pathways, Toll/IMD signaling, or other innate responses, thereby affecting the replication and dissemination of the secondary virus (Sim et al., 2014; Rückert et al., 2017).

Although DENV-1 and DENV-2 differ in their baseline vector competence, the sequential infection experiments were designed to characterize infection and transmission phenotypes following different orders of arbovirus acquisition. Therefore, comparisons between DENV-1 and DENV-2 within these groups describe serotype-specific patterns observed under sequential exposure and should not be interpreted as evidence of a differential CHIKV-mediated effect on the two DENV serotypes.

Sequential coinfection revealed serotype-specific differences in viral replication. When CHIKV was initiated first, DENV-1-infected mosquitoes exhibited higher body viral loads than DENV-2-infected mosquitoes at both 7 and 14 dpci. However, because DENV-1-infected mosquitoes also showed higher infectivity and body viral loads than DENV-2-infected mosquitoes under monoinfection conditions, these differences cannot be attributed exclusively to the interaction with CHIKV and may instead reflect intrinsic differences in the replication dynamics of the two dengue virus serotypes. Furthermore, despite the higher systemic viral loads observed for DENV-1, no corresponding differences were detected in salivary viral loads or transmission efficiency, indicating that greater body infection levels did not necessarily translate into enhanced transmission under the experimental conditions. Because the sequential infection design did not include sham-fed monoinfection controls, the relative contribution of intrinsic serotype biology and sequential coinfection could not be fully disentangled. Subsequent studies incorporating appropriate sequential control groups will help clarify the extent to which these factors contribute to the observed patterns.

Similarly, when DENV was introduced first, mosquitoes infected with DENV-1 had higher systemic and salivary viral loads than those infected with DENV-2 at both 7 and 14 dpci. Mosquitoes infected with DENV-1 also exhibited with higher viral titers in mouse ear tissues following mosquito bites and greater transmission efficiency than those infected with DENV-2. However, because DENV-1-infected mosquitoes also showed higher replication and transmission efficiency than those infected with DENV-2 under monoinfection conditions, these differences cannot be attributed exclusively to sequential coinfection with CHIKV, and may instead reflect intrinsic biological differences between the two dengue virus serotypes. Nevertheless, differences between DENV-1 and DENV-2 remained observable under the sequential exposure conditions evaluated here. Taken together, these findings suggest that intrinsic biological differences between DENV-1 and DENV-2 influence their replication and transmission dynamics during both monoinfection and sequential infections, while the contribution of CHIKV coinfection to these differences cannot be fully distinguished using the present experimental design (Vazeille et al., 2016; Fontaine et al., 2018).

When CHIKV was introduced before DENV infection, differences in CHIKV body viral loads were observed between mosquitoes subsequently challenged with DENV-1 and DENV-2 at both 7 and 14 dpci. Likewise, when DENV infection preceded CHIKV, differences in CHIKV salivary viral loads were observed between mosquitoes infected with the two dengue serotypes. Because the present analyses compared sequential infections initiated with DENV-1 or DENV-2, rather than comparing each sequential infection with the corresponding CHIKV monoinfection control, these findings should be interpreted as serotype-dependent differences within the sequential coinfection model rather than evidence that one serotype enhances or suppresses CHIKV vector competence relative to monoinfection. Future studies that include direct statistical comparisons with CHIKV monoinfection will be necessary to clarify the specific contribution of each DENV serotype to CHIKV replication and transmission dynamics.

The observation of distinct viral replication patterns across different sequential infection orders is consistent with previous studies demonstrating that arbovirus coinfection outcomes are highly context-dependent. In human hepatocyte-derived cells, prior CHIKV infection influenced subsequent DENV-2 replication, indicating that the outcome of sequential infections depends on the timing of infection and the cellular environment (Taraphdar et al., 2022). Likewise, Muturi and Bara (2015) demonstrated in C6/36 cells that prior Sindbis virus infection suppressed DENV-4 replication, supporting the concept that pre-established alphavirus infections can modify the replication of subsequently introduced flaviviruses. In mosquitoes, Magalhães et al. (2018) reported that previous ZIKV infection increased MAYV infection rates, further demonstrating that sequential arbovirus interactions may either enhance or suppress viral replication depending on the virus combination. Although our findings indicate that CHIKV replication differed between sequential infections involving DENV-1 and DENV-2, direct comparisons with previous mosquito studies remain limited. To our knowledge, the only comparable study is that of Vazeille et al. (2010), who demonstrated the simultaneous presence of DENV-1 and CHIKV in *Aedes albopictus* following sequential infection but did not quantify viral loads or transmission efficiency. Therefore, our study extends these observations by showing that sequential coinfections involving DENV-1 and DENV-2 produced distinct viral replication patterns, although the extent to which these differences reflect intrinsic serotype biology or possible interactions with CHIKV remains to be elucidated.

The analysis of CHIKV during sequential exposure revealed distinct replication and transmission-related phenotypes between groups involving DENV-1 and DENV-2. However, these differences cannot be attributed specifically to the DENV serotype included in each sequential exposure scenario, given the limitations of the present experimental design.

In our experimental framework there are several limitations that should be considered when interpreting our results. Specifically, the sequential coinfection experiments were designed to characterize infection and transmission phenotypes following different orders of DENV and CHIKV acquisition. However, the lack of matched sequential controls in which mosquitoes received a non-infectious blood meal followed seven days later by DENV-1 or DENV-2 limits our ability to directly evaluate the specific effect of prior CHIKV infection on DENV replication. Absolute levels of replication in DENV-1 and DENV-2 may be indicative of intrinsic differences between serotypes, and not differential interactions with CHIKV. Thus, lower levels of DENV-2 replication compared to DENV-1, regardless of the infection order, should not be taken as evidence of a stronger or weaker interaction with CHIKV. Ideally, the effect of CHIKV should be determined by comparing DENV replication in single infection and coinfection for each serotype and then determining whether the magnitude of the change induced by CHIKV is different between DENV-1 and DENV-2. Thus, the lack of these matched sequential controls is an important limitation for drawing definitive conclusions about serotype-specific modulation of DENV replication by CHIKV.

There are also other limitations to consider. All experiments were conducted under controlled laboratory conditions, whereas natural coinfections in wild *Aedes aegypti* populations occur in complex ecological, environmental, and host-related contexts that cannot be fully replicated in the laboratory. Additionally, we tested only one *Ae. aegypti* population and one viral strain per arbovirus. The generalizability of our findings to other mosquito populations and viral strains is therefore uncertain, as vector competence and virus–vector and virus–virus interactions may differ depending on the mosquito and viral genotypes (Lequime et al., 2020). Furthermore, transmission efficiency was measured only at one relatively late time point (14 dpi), potentially missing temporal variation in viral replication, dissemination and transmission over the extrinsic incubation period. Finally, while previous studies have provided evidence for the involvement of mosquito innate immune responses, tissue tropism, and cellular competition in arbovirus interactions, these mechanisms were not directly examined here. Future experiments incorporating matched sequential controls, multiple time points, additional mosquito populations and viral genotypes, and mechanistic approaches will therefore be important to determine whether CHIKV modulates DENV replication and transmission, to what extent it does so, and whether such effects differ between DENV serotypes.

Future research should therefore: (i) characterize mosquito innate immune pathways (RNAi, Toll, IMD, and JAK/STAT) during simultaneous and sequential coinfections; (ii) investigate serotype-specific features of DENV, such as replication kinetics and tissue tropism; (iii) evaluate vertical transmission under simultaneous and sequential coinfection conditions; (iv) examine multiple mosquito populations and viral strains to determine the generalizability of the observed interactions; (v) assess vector competence at multiple time points throughout the extrinsic incubation period; and (vi) integrate experimental and mathematical modeling approaches to investigate how within-vector viral interactions influence arbovirus transmission dynamics at the population level.

Although our experiments were performed under laboratory conditions, the distinct infection and transmission phenotypes observed across the sequential exposure groups may be relevant to regions where DENV and CHIKV cocirculate, such as Brazil. Because sequential exposure to different arboviruses may occur during outbreaks, characterizing viral phenotypes under different orders of virus acquisition may contribute to a better understanding of the heterogeneity in mosquito infection and transmission dynamics. However, extrapolation of these findings to field conditions should be made cautiously, as environmental factors, mosquito genetic diversity, viral genetic diversity, and host availability may substantially influence these outcomes.

Additional studies should prioritize the elucidation of the cellular and molecular mechanisms potentially underlying arbovirus interactions in *Aedes aegypti*. Arboviruses may compete for susceptible cell populations, host translational machinery, and intracellular resources required for replication, while also differentially activating innate immune pathways such as RNAi, Toll, IMD, and JAK/STAT (Ciota and Kramer, 2010; McFarlane et al., 2014; Sim et al., 2014; Moon et al., 2015; Rückert et al., 2017; Oliveira et al., 2020). Because these mechanisms were not directly investigated in the present study, it remains unclear whether intracellular competition, immune-mediated processes, or other mechanisms contribute to the observed phenotypes. These interactions should also be evaluated using multiple mosquito populations and viral strains, and at different time points following infection, to determine the generalizability and temporal dynamics of these effects. Finally, integrating experimental data with epidemiological modeling may help determine how sequential arbovirus infections influence transmission dynamics in regions where DENV and CHIKV cocirculate (**Supplementary Figure 2**).

## 5 Conclusion

Our findings demonstrate that simultaneous CHIKV and DENV coinfection and different orders of sequential arbovirus exposure are associated with distinct infection and transmission phenotypes in *Ae. aegypti*. These observations highlight the complexity of arbovirus coinfections within mosquito vectors and indicate that their outcomes may vary according to the DENV serotype and infection scenario.

During simultaneous coinfection, DENV-1 showed greater reductions in systemic infection and viral replication than DENV-2, while the sequential exposure experiments revealed distinct infection and transmission phenotypes between groups involving DENV-1- and DENV-2 and across different orders of virus acquisition. Although differences in salivary viral loads were observed under specific conditions, transmission efficiency was evaluated only at a single, relatively late time point (14 dpi). Therefore, the present study cannot exclude the possibility that coinfection may affect earlier stages of viral dissemination to saliva or the duration of the extrinsic incubation period.

The sequential infection experiments should, however, be interpreted within the limitations of the experimental design. Matched sequential controls in which mosquitoes received a non-infectious blood meal followed by DENV-1 or DENV-2 seven days later were not included. Consequently, the specific effect of prior CHIKV infection on each DENV serotype cannot be distinguished from other effects associated with sequential exposure. In addition, differences observed between DENV-1 and DENV-2 may reflect intrinsic differences in their vector competence and replication dynamics and should therefore not be interpreted as evidence of a differential CHIKV-mediated effect on the two serotypes.

Additional limitations should also be considered. The use of a single *Ae. aegypti* population and one viral strain per arbovirus limits the generalizability of the findings, and the cellular and molecular mechanisms underlying the observed patterns were not directly investigated. Thus, it remains unclear to what extent intracellular competition, mosquito immune responses, tissue tropism, or other processes contribute to the observed coinfection phenotypes.

Taken together, our findings characterize distinct infection and transmission phenotypes associated with simultaneous coinfection and different sequential arbovirus exposure scenarios under laboratory conditions. Future studies incorporating matched sequential controls, multiple mosquito populations and viral genotypes, additional transmission time points, and mechanistic approaches will be important to determine the specific contribution of virus–virus interactions to these phenotypes and establish whether the observed patterns extend to natural transmission settings where DENV and CHIKV cocirculate.

## Data Availability

The original contributions presented in the study are included in the article/**Supplementary Material**. Further inquiries can be directed to the corresponding author.
