# Supplementary material for: The consequence of simultaneous and sequential coinfections of Aedes aegypti with DENV-1, DENV-2, and CHIKV on vector competence
Source: Front Cell Infect Microbiol. 2026 Oct 1;16:1891568. doi: 10.3389/fcimb.2026.1891568 (PMC13668739; doi:10.3389/fcimb.2026.1891568)
Supplement: Supplementary file 1 [file Table1.docx]

**Supplementary Table 1.** Infection status of individual *Aedes aegypti* mosquitoes following simultaneous coinfection with DENV-1/CHIKV or DENV-2/CHIKV.

| **Coinfection group** | **Time** | **DENV+/CHIKV+** | **DENV+/CHIKV−** | **DENV−/CHIKV+** | **DENV−/CHIKV−** |
| --- | --- | --- | --- | --- | --- |
| DENV-1/CHIKV | 7 dpi | 25 (83.3%) | 1 (3.3%) | 4 (13.3%) | 0 (0%) |
|  | 14 dpi | 23 (76.7%) | 4 (13.3%) | 3 (10.0%) | 0 (0%) |
|  | 21 dpi | 29 (96.7%) | 1 (3.3%) | 0 (0%) | 0 (0%) |
| DENV-2/CHIKV | 7 dpi | 24 (80.0%) | 0 (0%) | 6 (20.0%) | 0 (0%) |
|  | 14 dpi | 22 (73.3%) | 5 (16.7%) | 2 (6.7%) | 1 (3.3%) |
|  | 21 dpi | 16 (53.3%) | 0 (0%) | 14 (46.7%) | 0 (0%) |
